# PAR1- and PAR2-induced innate immune markers are negatively regulated by PI3K/Akt signaling pathway in oral keratinocytes

**DOI:** 10.1186/1471-2172-11-53

**Published:** 2010-10-28

**Authors:** Maryam G Rohani, Dennis H DiJulio, Jonathan Y An, Beth M Hacker, Beverly A Dale, Whasun O Chung

**Affiliations:** 1 Department of Medicine/Dermatology, University of Washington, Seattle, WA, 98195-6524, USA; 2 Department of Oral Biology, University of Washington, Seattle, WA, 98195-7132, USA; 3 Department of Periodontics, University of Washington, Seattle, WA, 98195-7444, USA

## Abstract

**Background:**

Protease-Activated Receptors (PARs), members of G-protein-coupled receptors, are activated by proteolytic activity of various proteases. Activation of PAR1 and PAR2 triggers innate immune responses in human oral keratinocytes (HOKs), but the signaling pathways downstream of PAR activation in HOKs have not been clearly defined. In this study, we aimed to determine if PAR1- and PAR2-mediated signaling differs in the induction of innate immune markers CXCL3, CXCL5 and CCL20 via ERK, p38 and PI3K/Akt.

**Results:**

Our data show the induction of innate immunity by PAR1 requires both p38 and ERK MAP kinases, while PAR2 prominently signals via p38. However, inhibition of PI3K enhances expression of innate immune markers predominantly via suppressing p38 phosphorylation signaled by PAR activation.

**Conclusion:**

Our data indicate that proteases mediating PAR1 and PAR2 activation differentially signal via MAP kinase cascades. In addition, the production of chemokines induced by PAR1 and PAR2 is suppressed by PI3K/Akt, thus keeping the innate immune responses of HOK in balance. The results of our study provide a novel insight into signaling pathways involved in PAR activation.

## Background

Protease-activated receptors (PARs) are G-protein-coupled receptors (GPCRs) with a unique mechanism of activation. These receptors carry their own tethered ligands and are activated by proteolytic activity of serine proteases [1]. Among the four members of the PAR family, PAR1 and PAR2 are highly expressed in human oral keratinocytes (HOKs) [2]. PAR1 is activated by thrombin and PAR2 is activated by trypsin-like enzymes, including trypsin, mast cell tryptase and neutrophil proteinse-3 [3]. Activation of PARs by proteases of pathogens *Porphyromonas gingivalis *and *Aggregatibacter actinomycetemcomitans*, the Gram-negative bacteria associated with periodontitis, suggests a role for PARs and particularly PAR2 as a putative mediator of periodontitis [2,4-7].

Periodontitis is an infection of periodontal tissues which are the supportive structure for the teeth. In the complex structure of periodontal tissues, gingival epithelium is the first layer which encounters various periopathogens, acting as a physical barrier and playing an active role in innate immunity [8,9]. Oral keratinocytes utilize PAR1 and PAR2 as part of their ability to sense their environment, and activation of these receptors induces up-regulation of several cytokines, chemokines as well as antimicrobial peptides [2,10,11].

Findings from our earlier study showed that activation of PARs induced expression of CXCL3/MIP-2b, CXCL5/ENA-78 and CCL20/MIP-3α in HOKs [12]. CXCL3 and CXCL5 stimulate the chemotaxis of monocytes and neutrophils and both interact with the chemokine receptor CXCR2 [13]. CCL20 is strongly chemotactic for lymphocytes and dendritic cells and elicits its effect by activating chemokine receptor CCR6 [14]. These findings suggest that the major function of PAR1 and PAR2 in oral keratinocyte is to initiate and prolong innate immune responses via attraction of cells of the immune system such as leukocoytes and dendritic cells. Understanding the signaling pathways downstream of PAR1 and PAR2 activation leading to such responses will help us better understand how innate immune responses are regulated in maintaining oral health.

In the current work, we studied differential signaling of PAR1- and PAR2-mediated innate immune responses in the induction of CXCL3, CXCL5 and CCL20 via ERK, p38 and PI3K/Akt signaling. We hypothesized that the induction of these markers by PAR1 and PAR2 is differentially mediated by activation MAPK and PI3K, and used selective inhibitors for components of these signaling pathways to study their effects on PAR signaling. The results provide a novel insight into signaling pathways involved in PAR activation.

## Methods

### Primary HOKs isolation and cell culture

Tissue preparation and cell culture method for primary HOKs have been described previously in detail [9]. Briefly, healthy gingival tissue samples from patients undergoing third molar extraction were collected for tissue culture with patients' informed consent and according to the procedures approved by University of Washington Institutional Review-Board. Tissue samples were processed to dissociate the epithelium into single cells. For experiments, cells were grown in supplemented serum-free keratinocyte basal medium (KBM) (Cambrex, Walkersville, MD) and incubated at 37°C in 5% CO_2_. Fourth passage cells at 75-80% confluence were used for all experiments. Due to the possible variation between individual donors, we looked for consistent results in HOKs from at least three donors with technical duplicate for each set of experiments, unless otherwise stated.

### Reagents used

Human alpha-thrombin (Haematologic Technologies Inc, Essex Junction,VT) and recombinant human trypsin (Polymun Scientific Immunobiologische Forschung GmbH, Austria) were used to stimulate HOKs in order to activate PAR1 and PAR2, respectively. D-Phe-Pro-Arg-chloromethyl ketone dihydrochloride (PPACK-HCL, Calbiochem, La Jolla, CA) and serine protease inhibitor, tosyl-L-lysine chloromethyl ketone (TLCK, Sigma, St. Louis, MO) were used to inhibit thrombin and trypsin, respectively. Inhibitors for ERK1/2 (U0126) and its control substance (U0124), p38 (SB203580), PI3K (Wortmannin and LY294002), Akt (Akt inhibitor IV) were from Calbiochem (La Jolla, CA). Rabbit polyclonal p38 MAPK (9212) and Akt (9272), and monoclonal phospho-p38 MAPK (Thr180/Tyr182; D3F9), phospho-Akt (Thr308; 244F9), p44/p42 MAPK (ERK1/2; 137F5), and phospho-p44/p42 MAPK (Thr202/Tyr204;D13.14.4E, XP™) antibodies were obtained from Cell Signaling Technology, Inc. (Danvers, MA). Mouse monoclonal GAPDH, used as western blot control for equal gel load of protein, was purchased from Santa Cruz Biotechnology Inc. (Santa Cruz, CA). As assessed by the Limulus Amebocyte Lysate Pyrotell (Cape Cod Inc., Falmouth, MA), the thrombin (1 U/ml) and trypsin (1 nM) preparations in our study contained less than 0.03 EU LPS/ml.

### RNA isolation, reverse transcription and Quantitative RT-PCR (QRT-PCR)

Single-stranded cDNA was synthesized from total RNA and used to perform QRT- PCR with gene-specific primers as described previously [6,12]. Unstimulated HOKs and samples without RT served as negative controls. A melting curve was performed at the end of each QRT-PCR to ensure the gene product was specific. Glyceraldeyde-3-phosphate dehydrogenase (GAPDH) was used as the selected housekeeping gene for normalization. The primer sequences for GAPDH, CXCL5, CXCL3 and CCL20 have been described previously [6,12,15]. Data were analyzed using Pfaffl method to calculate fold change of gene expression [16].

### Measurement of chemokines in culture supernatant

Secreted CXCL5 and CCL20 were measured in culture supernatant using Duoset ELISA kit (R&D, Minneapolis, MN). The absolute concentration in each sample was calculated based on sample yielding optical density using the standard curve. Chemokine secretion from each condition was normalized to chemokine produced at baseline level in unstimulated control group, and results are presented as percent of unstimulated control cells.

### ELISA assay for quantification of p38 and ERK1/2 phosphorylation

To assess phosphorylation of p38 and ERK1/2, treated cells were homogenized in lysis buffer in accordance with the manufacturer's protocol (R&D). Immediately prior to adding to cells, protease and phosphatase inhibitors, PMFS and sodium orthovanadate (Santa Cruz Biotechnology), Halt Protease Inhibitor Cocktail and Halt Phosphatase Inhibitor Cocktail (Pierce, Rockford, IL), were added to the lysis buffer. Phosphorylation of p38 and p42/44 (ERK1/2) was assessed in cell lysate using Duoset antibody pairs following manufacturer's protocol (R&D). Total p38 was quantified by a similar method (R&D) and served for normalization. Total p38 was proportional to total protein measured in each sample and to the number of cells plated in each well.

### Western blot analysis

Total and phosphorylated kinase expression in untreated and agonist-stimulated HOK cells was determined by Western immunoblot analysis. Samples of whole cell lysate supernatant proteins, solubilized in 1X NuPage LSD sample and reducing buffer, were resolved concomitantly with Precision Plus Dual Color protein molecular weight standard (Bio-Rad, Hercules, CA) in NuPAGE 4-12% Bis-Tris mini gel (1.5 mm, 10 well) and MOPS SDS running buffer in XCell SureLock Mini-Cell, transferred to PVDF membrane (Immobilon-P, Millipore, Chicago, Il) in XCell II Blot Module using electrophoresis systems and protocols of InVitrogen Corp. (Carlsbad, CA). PVDF membranes were blocked for 1 h with 1% BSA, 1%PVP and 1% PEG in wash buffer (0.1% Tween-20, 100 mM Tris/HCl, 20 mM NaCl), then: (1) incubated with rabbit primary antibodies either overnight at 4°C for phospho-Akt antibody, or 1 h at room temperature for total (Akt, p38 MAPK and p44/p42 MAPK) and phospho- proteins (p38 MAPK, and p44/p42 MAPK) at concentrations recommended by the manufacturer, then incubated for 1 h with donkey anti-rabbit IgG (H+L) HRP conjugate (Jackson ImmunoResearch, West Grove, PA) diluted 1:20,000 in 0.1% Tween-20, Tris Buffered Saline (TBS); or (2) incubated with mouse monoclonal GAPDH diluted 1:5,000 in blocking buffer and then with goat anti-mouse IgG (H+L) HRP conjugate (JacksonImmunoResearch) diluted 1:50,000 in 0.1%TTBS. Secondary antibody binding to primary antibody was detected using SuperSignal West Dura Extended Duration chemiluminescent substrate and protocols of Thermo Scientific (Rockford, IL). Film exposures were chosen to give unsaturated blot densities. GAPDH immunoblots showing equal gel load of protein were obtained without stripping. Total protein immunoblots for Akt, p38 MAPK and p44/p42 MAPK were obtained from separate gels loaded with 2 to 5 fold less protein. Chemiluminograms of immunoblots were digitally scanned and saved in TIFF format using Photoshop™ software (Adobe System Inc., San Jose, CA) without further processing.

### Statistical analysis

Data collected from at least three donors are presented as mean ± standard error of the mean (S.E.M). Data were compared using one-way ANOVA followed by Bonferroni post test. A p-value < 0.05 was considered to be significant.

## Results

### Activation of PARs modulates phosphorylation of ERK1/2 and p38 in primary HOKs

**Figure 1:**
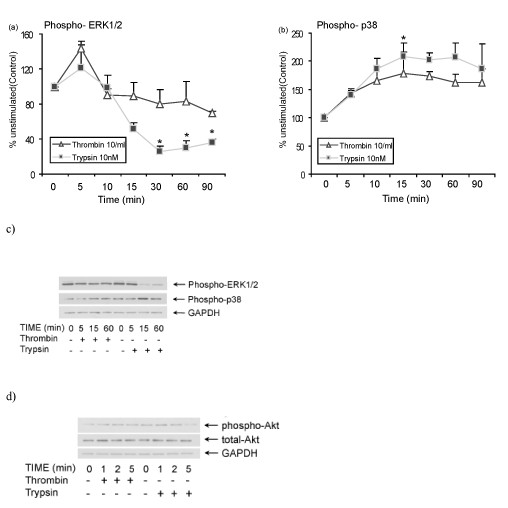
**The effect of PAR activation on the kinetics of ERK1/2, p38 and Akt phosphorylation**. HOKs were stimulated with thrombin (10 U/ml) or trypsin (10 nM) for 0-90 min. (a-b) Phosphorylation of ERK1/2 and p38 was quantified in cell lysates using ELISA. Data are given as percent of unstimulated cells and as means ± S.E.M from two different donors. (*p < 0.05 compared to unstimulated control at 0 min). (c-d) The Western blots analysis of whole cell lysates from cells stimulated with thrombin (10 U/ml) or trypsin (10 nM) at indicated times. Cell lysates were analyzed by immnoblotting with specific antibodies to phospho-ERK1/2, total ERK1/2, phospho-p38, total p38, phospho-Akt and total Akt. GAPDH was served as a control to verify equal loading and transfer of samples.

We examined the dynamic activation of ERK1/2 and p38 when cells were stimulated with thrombin and trypsin. ELISA-based analysis showed a transient weak and rapid (5 min) phosphorylation of ERK1/2 subsequent to both PAR1 and PAR2 activation. In PAR1-activated cells, the level of ERK1/2 activity was at its maximum within 5 min and then decreased and remained at a steady level close to baseline up to 90 min. In contrast, after its maximum phosphorylation at 5 min, PAR2 activation induced dephosphorylation of ERK1/2 to below the baseline level for up to 90 min (Figure 1a). Both PAR1 and PAR2 activation also induced phosphorylation of p38 within 5 min (Figure 1b). The activation of p38 increased steadily and reached a maximum at 15-30 min. However, PAR2 activation resulted in a higher level of p38 phosphorylation than with PAR1 activation (Figure 1b). These results were confirmed by Western immunoblot analysis as well. Neither PAR1 nor PAR2 was a strong inducer of ERK1/2 phosphorylation, either at times shorter than 5 min or between 5-60 min after stimulation (Figure 1c). A striking change in phospho-ERK1/2 is induced by PAR2 activation, which causes dephosphorylation of phospho-ERK1/2 in 15 min. In accordance with ELISA results, both PAR1 and PAR2 activation induced p38 phosphorylation, which was sustained up to 60 min (Figure 1c, second row). We next tested the effect of PAR1 and PAR2 activation on phorphorylation of Akt (also known as protein kinase B, PKB). Akt is a serine/threonine protein kinase and activated by stimuli that induce production of phosphatidylinositol (3,4,5)-trisphosphate via activation of PI3K [17]. Results show a rapid phosphorylation of Akt in effect of PAR1 and PAR2 activation (Figure 1d). These results suggest involvement of MAP kinases and PI3K/Akt in cellular signaling downstream of PAR activation and identify distinct patterns of ERK1/2 and p38 MAPK phosphorylation by PAR1 and PAR2. Phosphorylation of ERK1/2 was subtle and transient, while p38 phosphorylation was prolonged. The kinetic analysis suggests ERK1/2 is more involved in PAR1 signaling, while p38 has greater participation in PAR2 signaling.

### The innate immune markers induced by PAR1 and PAR2 activation are regulated by ERK1/2 and p38 MAPK

**Figure 2:**
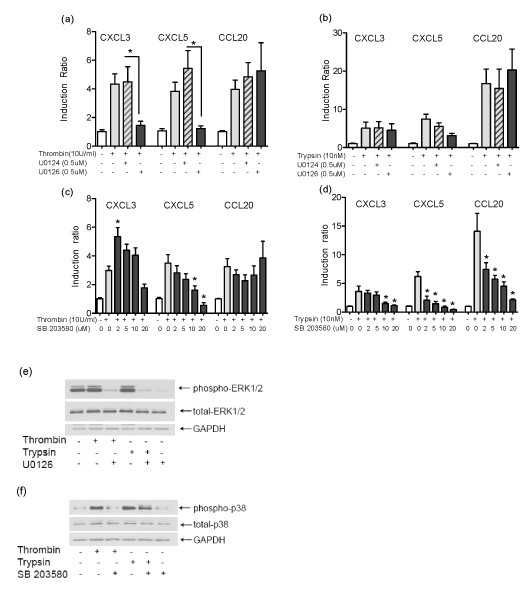
**PAR1 and PAR2 signal via ERK1/2 and p38 MAP kinases to induce expression of innate immune markers**. (a-b) HOKs were incubated with a selective inhibitor for ERK1/2 and its control substance, U0126 and U0124, respectively, at 500 nM or (c-d) a p38 inhibitor, SB203580 at 2-20 μM for 1 h, then stimulated with thrombin (10 U/ml) or trypsin (10 nM) for 6 h. The mRNA expression of CXCL3, CXCL5 and CCL20 was measured by QRT-PCR. Data from three different donors set up in duplicates were normalized to GAPDH. Data are given as means of normalized samples to unstimulated control ± SEM. (*p < 0.05 compared to the same condition without inhibitors). (e-f) To test the efficacy of inhibitors, HOKs were incubated with U0126 (0.5 μM) or SB203580 (20 μM) for 1 h, and then stimulated with thrombin (10 U/ml) for 2 min or trypsin (10 nM) for 15 min. The Western blot analysis was done using antibodies specific for phospho- ERK1/2, total-ERK1/2, phospho-p38 and total-p38. GAPDH was used as a control for equal loading of samples.

In our previous studies we found that thrombin induced CXCL3 and CXCL5 via PAR1, while trypsin induced up-regulation of CXCL3, CXCL5 and CCL20 via PAR2 activation [12]. In this study, we investigated the signaling molecules involved in the induction of these innate immune markers following PAR1 and PAR2 activation in HOKs. As activation of PAR1 and PAR2 modulates phosphorylation of p38 and ERK1/2, next we analyzed the role of ERK1/2 and p38 in the PAR1- and PAR2-induced CXCL3, CXCL5 and CCL20 mRNA expression. Inhibition of ERK1/2 by U0126, which inhibits the signaling molecule upstream of ERK1/2, significantly blocked the expression of CXCL3 and CXCL5 induced by PAR1 activation, but had no significant effect on the induction of the three markers by PAR2 activation (Figure 2a-b). Inhibition of p38 by SB203580 had a stimulatory effect at low concentration (2-10 μM) on PAR1-induced CXCL3, but the effect was attenuated at higher concentration (20 μM). In the presence of the p38 inhibitor, PAR1-activated cells showed a decrease in CXCL5 expression in a dose-dependent manner but there was no effect on CCL20 expression (Figure 2c). In contrast, induction of all three markers by PAR2 activation was significantly blocked by the p38 inhibitor in a dose-dependent manner (Figure 2d). The inhibitors on their own did not affect the expression of the selected markers (data not shown). Furthermore, the efficacy of the inhibitors was tested. Immunoblot analysis showed a reduction in phosphorylation of ERK1/2 and p38 in the presence of U0126 and SB203580, respectively (Figure 2e-f). These results suggest that both ERK1/2 and p38 are activated downstream of PARs signaling to induce appropriate innate immune responses. Expression of the selected markers of innate immunity induced by PAR1 activation is more dependent on ERK1/2. In contrast, PAR2 signaling is more dependent on p38 in the induction of innate immune responses.

### Innate immune expression in response to PAR1 and PAR2 activation is enhanced by PI3K/Akt inhibition

**Figure 3:**
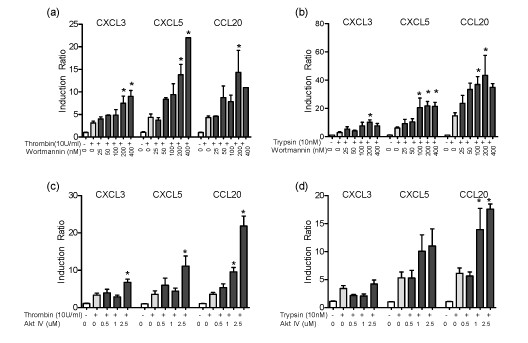
**Expression of PAR1- and PAR2-induced innate immunity is enhanced when PI3K/Akt signaling pathway is blocked**. HOKs were incubated with inhibitors for PI3K (Wortmannin) at 25-400 nM (a-b) or for Akt (Akt inhibitor IV) at 0.5-2.5 uM (c-d) for 1 h and subsequently stimulated with thrombin (10 U/ml) or trypsin (10 nM) for 6 h. Cells were harvested and expression of the selected markers was assessed by QRT-PCR. Data from three separate donors set up in duplicates were normalized to GAPDH. Data are given as means of normalized samples to unstimulated control ± SEM. (*p < 0.05 compared to the same condition without inhibitors).

The PI3K/Akt signaling pathway plays a role in coordinating defense mechanisms in innate immunity [18,19]. Increased phosphorylation of Akt suggested activation of PI3K/Akt pathway downstream of PARs. In order to determine its role in the regulation of selected innate immune markers mediated via PAR1 and PAR2, we used selective inhibitors for PI3K. Inhibition of PI3K by two specific inhibitors, Wortmannin (Figure 3a-b) and LY294002 (data not shown), induced a concentration-dependent enhancement of CXCL3, CXCL5 and CCL20 mRNA expression in PAR1- and PAR2-activated cells, thus suggesting that PI3K has an inhibitory effect on innate immune responses induced by both PAR1 and PAR2. In order to confirm this negative regulatory effect of PI3K, we tested the effect of blocking Akt on responses induced by PAR1 and PAR2 activation. Blocking Akt activity by Akt inhibitor IV, which inhibits a kinase upstream of Akt but downstream of PI3K, also resulted in an increase in expression of all three markers induced by PAR1 at higher doses of inhibition, and increased CCL20 expression induced by PAR2 activation (Figure 3c-d). These results suggest that the PI3K/Akt signaling pathway limits the innate immune responses activated by PAR1 and PAR2.

### Secretion of CXCL5 in response to PAR1 and PAR2 activation is enhanced by PI3K/Akt inhibition

**Figure 4:**
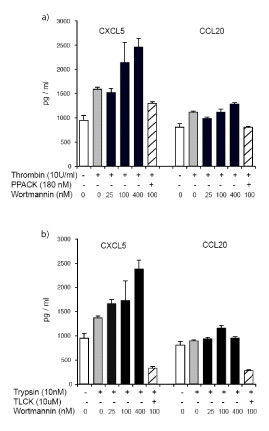
**PAR-induced secretion of CXCL5 is increased when PI3K is inhibited**. HOKs were incubated with Wortmannin at 25-400 nM for 1 h. Then cells were stimulated with thrombin (10 U/ml), trypsin (10 nM) or inactivated form of thrombin and trypsin by using PPACK (180 nM) and TLCK (10 μM), respectively, for additional 1 h. Subsequently cells were washed and incubated in fresh medium for 24 h. CXCL5 and CCL20 were measured in the culture supernatant using ELISA. Data are representative of two separate experiments using cells from different donors.

A previous study reported inhibitory effect of PI3K signaling pathways following activation of Toll-like receptor 4 by LPS [20]. In our studies we confirmed no endotoxin contamination in thrombin and trypsin. However, in order to exclude the possibility that endotoxin contamination of inhibitors may be responsible for increased expression of innate immune markers, and also to test if increased induction of selected markers is associated with the secretion of mature proteins, we measured CXCL5 and CCL20 in culture supernatant when cells are stimulated with thrombin and trypsin for PAR1 and PAR2 activation, respectively, or with the inactivated form of the enzymes in the presence of PI3K inhibitor. CXCL5 secretion induced by PAR1 activation was increased when PI3K activity was inhibited, and this effect was abrogated in the presence of PPACK to block thrombin proteolysis. A similar pattern was observed for secreted CXCL5 induced by PAR2 activation, and the effect was abrogated in the presence of TLCK to inhibit trypsin. However, secreted level of CCL20 did not change significantly in the presence of the PI3K inhibitor in either PAR1- or PAR2-activated cells (Figure 4a-b). Taken together, our data suggest that PI3K is a negative regulator of innate immune markers induced by activation of PAR1 and PAR2.

### Inhibition of PI3K is associated with decreased ERK1/2 and increased p38 phosphorylation

**Figure 5:**
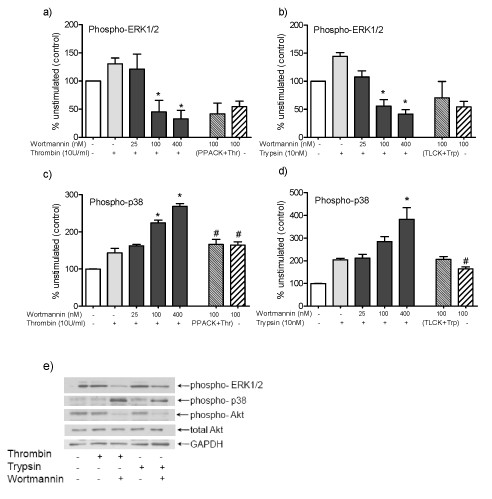
**Inhibition of PI3K is associated with decreased ERK1/2 and increased p38 phosphorylation**. HOKs were pretreated with 25-400 nM Wortmannin for 1 h followed by stimulation with (a,c) thrombin (10 U/ml) or (b,d) trypsin (10 nM). Phospho ERK1/2 at 5 min and phospho p38 at 15 min after PAR1/PAR2 activation were quantified in cell lysate by ELISA. Data are given as percent of unstimulated cells and as means ± S.E.M from two different donors. (*p *< 0.05, * vs. cells treated with thrombin or trypsin in the absence of inhibitors, # vs. Wortmannin 100 nM+ (thrombin or trypsin)). (e) For immunoblot analysis of phospho-ERK1/2 and phospho-p38, HOKs were preincubated with Wortmannin (400 nM) for 1 h followed by stimulation with thrombin (10 U/ml) or trypsin (10 nM). By using specific antibodies for phosphor-ERK1/2, total ERK1/2, phosphor-p38 and total-p38, phosphorylation of ERK1/2 and p38 was evaluated at 5 min and 15 min, respectively. To test the efficacy of Wortmannin in blocking PI3K/Akt pathway, above sample lysates were analyzed by immunoblotting using antibodies specific for phospho-Akt and total-Akt. GAPDH was used as a control for equal loading of samples.

Since activation of MAPK and PI3K signal transduction had opposite effects on innate immune responses induced by PAR activation in HOKs, we hypothesized that PI3K has inhibitory effect on activation of ERK1/2 and p38 downstream of PAR1 and PAR2 signaling. We assayed the phosphorylation of ERK1/2 at 5 min and p38 at 30 min (the time of maximum phosphorylation of ERK1/2 or p38) when PI3K activity was inhibited by Wortmannin at various concentration and cells were stimulated with thrombin or trypsin for PAR activation. ELISA-based assay suggested that in these conditions, inhibition of PI3K by Wortmannin followed by PAR1 or PAR2 activation caused decreased phosphorylation of ERK1/2 in a dose-dependent manner (Figure 5a-b). In contrast, inhibition of PI3K increased phosphorylation of p38 in response to PAR activation, and these effects were correlated with increased concentration of PI3K inhibitors (Figure 5c-d). Inhibition of PI3K by LY294002 had similar effects as Wortmannin on cells activated with trypsin, but had less potent effects on cells activated with thrombin (data not shown). These findings were confirmed by Western immunoblot analysis as well. As shown in Figure 5e, inhibition of PI3K activity by Wortmannin decreased phosphoylation of ERK1/2, but increased p38 phosphorylation when PAR1 and PAR2 are activated. Moreover, the efficacy of Wortmannin in inhibition of PI3K is shown by decreased Akt phosphorylation, downstream of PI3K (Figure 5e). These results suggest that PAR1 and PAR2 activation leads to a crosstalk between activation of PI3K, ERK1/2 and p38, and that inhibition of PI3K results in decreased activation of ERK1/2 but increased activation of p38 downstream of PAR signaling.

## Discussion

The transmission of signals from cell membrane into the nucleus requires coordinated action of diverse signaling proteins. In this study we identified the key signaling molecules involved in the induction of innate immunity in human oral keratinocytes in response to PAR1 and PAR2 activation. PAR1 and PAR2 have been demonstrated to activate members of the MAPK signaling cascade in the induction of IL-8 and IL-1b in epithelial cells from different tissue origin [21-24]. In agreement with these reports, our findings indicated both p38 and ERK1/2 were phosphorylated by PAR1 and PAR2 activation. Our findings further reveal that the induction of additional innate immune markers, CXCL3, CXCL5 and CCL20, upon activation of PAR1 and PAR2 signals via p38 and ERK1/2. However, we observed divergent role for ERK1/2 and p38 MAPK in transducing signals for innate immunity by PAR1 and PAR2. PAR1 signals via both p38 and ERK1/2, whereas the induction of similar chemokines by PAR2 is primarily via p38. We also showed that PI3K activation had a negative regulatory role for both PAR1 and PAR2 signaling and thus may limit proinflammatory responses induced by proteases in the environment.

Our kinetics studies demonstrated transient ERK1/2 phosphorylation by PAR1 and PAR2 activation, which was followed by a distinct pattern of ERK1/2 dephosphorylation. This was more prominent with PAR2 activation compared to PAR1 activation. This may explain the minimum effect of inhibition of ERK1/2 for PAR2-mediated innate immune responses. On the other hand, PAR2 activation, compared to PAR1, resulted in more effective phosphorylation of p38. These data suggest that dephosphorylation of ERK1/2 following PAR2 activation may be a protective mechanism against excess innate immune responses via p38 and ERK1/2. A similar protective effect by down-regulation of MAPK signaling downstream of PAR2 activation is reported in acute pancreatitis induced by an intraperitoneal injection of caerulein in rats [25]. However, the mechanism of ERK dephosphorylation by PAR2 activation is still unclear, and we are investigating whether PAR2 signaling mediates activation of phosphatases or if other mechanisms are involved.

Inhibition of p38 also differentially affected the expression of selected markers induced by PAR1 and PAR2 activation with different sensitivity to the presence of inhibitor for each marker. This may be related to the involvement of different p38 subunits (α,β,γ,δ) with differential downstream signaling and also to the lack of the equipotency of the current inhibitor against all subunits [26].

Our studies suggest PI3K has an inhibitory effect on PAR signaling in HOKs. This effect was shown most clearly at the mRNA level and also for CXCL5 at protein level. We did not observe this effect in the secretion of CCL20, which may be related either to the peptide structure of CCL20 which is vulnerable to proteolytic activity of enzymes, or to involvement of other mechanisms that affect CCL20 expression at the post-transcriptional level. Little information is available about PAR-mediated PI3K signaling in normal human keratinocytes with comparable cellular function, but our results indicate HOKs have a unique signaling system. It has been shown that thrombin signals via PI3K to induce osteoprotegerin in human periodontal ligament and VEGF in human pigment retinal epithelial cells [23,27]. In a recent study Minhajuddin *et al*. showed that PI3K/Akt is involved in modulation of NF-κB and expression of ICAM-1 induced by thrombin in endothelial cells. Their study suggested that activation of PI3K/Akt leads to activation of mTOR. While the over-expression of the catalytic domain of Akt increases activation of NF-κB in the absence of mTOR activity, restoring mTOR signaling dampens activation of NF-κB and induction of ICAM-1 [28]. In an earlier study by this group it was reported that thrombin-mediated ICAM-1 induction relies on parallel activation of PI3K and PKC that converges at Akt and induces activation of NF-κB [29]. In contrast to their findings, our results suggest a direct inhibitory role for PI3K/Akt. These discrepancies may be explained by cell type-specific signaling mechanisms or different markers that have been investigated. Our finding in HOKs suggests PI3K acts as a compensatory mechanism which suppresses inflammatory responses. A similar inhibitory role for PI3K signaling in response to TLR2 and TLR5 activation has been reported in monocytes, dendritic cells and epithelial cells [30-32], suggesting that PI3K may act as a balancing point to prevent excessive innate immune responses. It has been reported that PI3K knockout mice compared to their heterozygous littermates displayed increased levels of IL-6, IL-8 and nitrite in response to TLR5 activation [32]. Results from our study suggest that inhibition of PI3K/Akt resulted in the up-regulation of innate immune markers CXCL3, CXCL5 and CCL20 via PAR activation in HOKs.

Our results suggest that the mechanism of crosstalk between PI3K and PAR signaling is via effect on phosphorylation of p38 and ERK1/2. We observed inhibition of PI3K resulted in increased p38 phosphorylation even in the absence of external stimulants (thrombin and tryspin), and this effect was significantly greater when cells were stimulated with active enzyme versus inactive form of thrombin and trypsin. This finding suggested a specific role of PAR activation in the induction of a crosstalk between PI3K and p38. This interaction between p38 and PI3K signaling pathways downstream of PARs activation may serve as a protective strategy HOKs utilize to keep innate immune responses in balance. Activation of PI3K inhibits the induction of proinflammatory chemokines possibly by suppression of p38 MAPK activation. When TLR5 is activated by flagellin in intestinal cells [32] and in VEGF-induced tissue factor in endothelial cells [33], suppressive effect of PI3K has been observed. Although we expected to see a similar relationship between ERK1/2 and PI3K activation, our studies showed blocking PI3K limited ERK1/2 activity and suggest that PI3K and ERK signaling pathways are acting in series. Other studies showed that inhibition of PI3K induced phosphorylation of ERK1/2 in intestinal epithelial cells stimulated with flagellin [32] and in hepatic stellate cells activated with platelet-derived growth factor [34]. These studies may reflect that the interaction between PI3K and ERK signaling varies depending on the stimulus and cell type.

Our studies suggested that PAR1 signals via both ERK1/2 and p38, but that ERK1/2 has a more prominent role. However, there was no role for either p38 or ERK1/2 in the induction of CCL20 by PAR1 activation, although its expression was increased when PI3K and Akt were inhibited. It is likely that an alternative mechanism, which is independent of the ERK1/2 and p38 pathways, but still blocked by PI3K, is involved in the induction of CCL20 by PAR1 activation. This is consistent with our previous study showing that CCL20 induction by thrombin may occur via a mechanism other than PAR1 [12].

Induction of cytokines and chemokines by PAR activation leads to infiltration of mononuclear cells in the microenvironment of periodontal tissue [5]. This process is part of the initial recognition of danger in the environment and serves as an important protective function. While this primary immune response can protect the body against pathogenic factors, over-activity of these responses can become destructive and lead to progressive diseases. In periodontal diseases, exaggerated immune responses lead to excess inflammation, thus it is potentially important that oral keratinocytes keep immune responses in balance by shutting down the expression of proinflammatory genes. It is likely that crosstalk between p38-MAPK and PI3K/Akt signaling pathways plays a role in this process. Downstream of PAR activation, PI3K has a suppressive effect on the regulation of chemokines, thus may act to minimize the potential negative consequences of over-activity of inflammatory responses. However, bacterial pathogens with ability to activate PAR could take advantage of this mechanism in gingival epithelium and dampen innate immune responses to increase the survival of pathogens, which will result in sustained infection. Thus, it is necessary to consider both sides of the role of PI3K/Akt in evaluating possible therapeutic targets. Furthermore, understanding the molecular events associated with PAR signaling in keratinocytes may open new possibilities of intervention for mucosal inflammation such as periodontal diseases.

## Conclusion

**Figure 6:**
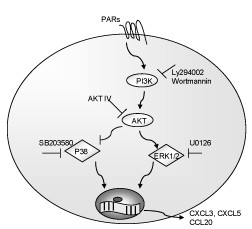
**PAR1 and PAR2 signaling involves MAPK and PI3K signaling pathways in the induction of innate immune responses**. Protease-activated receptors are G-protein-coupled receptors. Once activated, they signal via ERK1/2 and p38 MAPK to induce innate immune responses. This action is limited by activation of PI3K-Akt signaling pathway. This inhibitory effect is predominantly via blocking of p38 activation.

We demonstrated in this study that the induction of inflammatory responses by PAR1 and PAR2 is differentially regulated by ERK1/2 and p38 MAPK signaling pathways. ERK1/2 and p38 are both involved in signaling via PAR1, but p38 is more critical for signaling via PAR2. PI3K has a negative regulatory role limiting proinflammatory gene expression induced by both PAR1 and PAR2. We characterized crosstalk between PI3K/Akt and MAPK signaling pathways and the possibility of p38 phosphorylation as one of the mechanisms by which PI3K keeps innate immune responses in balance subsequent to PAR activation. A simple schematic overview of PAR signaling is summarized in Figure 6.

## Abbreviations

PAR: protease-activated receptor; HOK: human oral keratinocyte; PPACK: D-Phe-Pro-Arg-chloromethyl ketone; TLCK: tosyl-L-lysine chloromethyl ketone;

## Authors' contributions

MGR designed experiments, performed QRT-PCR and ELISA, and drafted the manuscript. DHD and JYA stimulated cells and conducted Western blot analyses. BMH performed GECs culture. BAD oversaw experimental design and helped drafting of the manuscript and discussion. WOC oversaw experimental design, contributed to discussion, drafting and revision of the manuscript. All authors read and approved the final manuscript.

## References

[B1] Coughlin SR Thrombin signalling and protease-activated receptors Nature 2000 407 6801 258 64 10.1038/35025229 11001069

[B2] Lourbakos A Arginine-specific protease from Porphyromonas gingivalis activates protease-activated receptors on human oral epithelial cells and induces interleukin-6 secretion Infect Immun 2001 69 8 5121 30 10.1128/IAI.69.8.5121-5130.2001 11447194 PMC98608

[B3] Coughlin SR Camerer E PARticipation in inflammation J Clin Invest 2003 111 1 25 7 12511583 10.1172/JCI17564 PMC151847

[B4] Holzhausen M Spolidorio LC Vergnolle N Role of protease-activated receptor-2 in inflammation, and its possible implications as a putative mediator of periodontitis Mem Inst Oswaldo Cruz 2005 100 Suppl 1 177 80 15962119 10.1590/s0074-02762005000900030

[B5] Holzhausen M Spolidorio LC Vergnolle N Proteinase-activated receptor-2 (PAR2) agonist causes periodontitis in rats J Dent Res 2005 84 2 154 9 10.1177/154405910508400209 15668333

[B6] Dommisch H Protease-activated receptor 2 mediates human beta-defensin 2 and CC chemokine ligand 20 mRNA expression in response to proteases secreted by Porphyromonas gingivalis Infect Immun 2007 75 9 4326 33 10.1128/IAI.00455-07 17591792 PMC1951157

[B7] Shimada T Protease-activated receptor 2 mediates interleukin-8 and intercellular adhesion molecule-1 expression in response to Aggregatibacter actinomycetemcomitans Oral Microbiol Immunol 2009 24 4 285 291 10.1111/j.1399-302X.2009.00507.x 19572889

[B8] Dale BA Fascination with epithelia: architecture, proteins, and functions J Dent Res 2003 82 11 866 9 10.1177/154405910308201104 14578496

[B9] Chung WO Dale BA Innate immune response of oral and foreskin keratinocytes: utilization of different signaling pathways by various bacterial species Infect Immun 2004 72 1 352 8 10.1128/IAI.72.1.352-358.2004 14688115 PMC343965

[B10] Chung WO Protease-activated receptor signaling increases epithelial antimicrobial peptide expression J Immunol 2004 173 8 5165 70 15470061 10.4049/jimmunol.173.8.5165

[B11] Uehara A Activation of human oral epithelial cells by neutrophil proteinase 3 through protease-activated receptor-2 J Immunol 2002 169 8 4594 603 12370398 10.4049/jimmunol.169.8.4594

[B12] Rohani MG Modulation of Expression of Innate Immunity Markers CXCL5/ENA-78 and CCL20/MIP3{alpha} by Protease Activated Receptors (PARs) in Human Gingival Epithelial Cells Innate Immun 2009 in press 19567485 10.1177/1753425909339233 PMC2846212

[B13] Mestas J The role of CXCR2/CXCR2 ligand biological axis in renal cell carcinoma J Immunol 2005 175 8 5351 7 16210641 10.4049/jimmunol.175.8.5351

[B14] Schutyser E Struyf S Van Damme J The CC chemokine CCL20 and its receptor CCR6 Cytokine Growth Factor Rev 2003 14 5 409 26 10.1016/S1359-6101(03)00049-2 12948524

[B15] Okada M Detection of up-regulated genes in thrombin-stimulated human umbilical vein endothelial cells Thromb Res 2006 118 6 715 21 10.1016/j.thromres.2005.11.008 16356540

[B16] Pfaffl MW A new mathematical model for relative quantification in real-time RT-PCR Nucleic Acids Res 2001 29 9 e45 10.1093/nar/29.9.e45 11328886 PMC55695

[B17] Paez J Sellers WR PI3K/PTEN/AKT pathway. A critical mediator of oncogenic signaling Cancer Treat Res 2003 115 145 67 full_text 12613196

[B18] Gunzl P Schabbauer G Recent advances in the genetic analysis of PTEN and PI3K innate immune properties Immunobiology 2008 213 9-10 759 65 10.1016/j.imbio.2008.07.028 18926291

[B19] Weichhart T Saemann MD The PI3K/Akt/mTOR pathway in innate immune cells: emerging therapeutic applications Ann Rheum Dis 2008 67 Suppl 3 iii70 4 10.1136/ard.2008.098459 19022819

[B20] Fukao T Koyasu S PI3K and negative regulation of TLR signaling Trends Immunol 2003 24 7 358 63 10.1016/S1471-4906(03)00139-X 12860525

[B21] Yoshida N Interleukin-8 production via protease-activated receptor 2 in human esophageal epithelial cells Int J Mol Med 2007 19 2 335 40 17203209 10.3892/ijmm.19.2.335

[B22] Fyfe M PAR-2 activation in intestinal epithelial cells potentiates interleukin-1beta-induced chemokine secretion via MAP kinase signaling pathways Cytokine 2005 31 5 358 67 10.1016/j.cyto.2005.06.004 16095910

[B23] Bian ZM Elner SG Elner VM Thrombin-induced VEGF expression in human retinal pigment epithelial cells Invest Ophthalmol Vis Sci 2007 48 6 2738 46 10.1167/iovs.06-1023 17525207 PMC2128055

[B24] Wang L Induction of interleukin-8 secretion and activation of ERK1/2, p38 MAPK signaling pathways by thrombin in dermal fibroblasts Int J Biochem Cell Biol 2006 38 9 1571 83 10.1016/j.biocel.2006.03.016 16697690

[B25] Namkung W PAR2 exerts local protection against acute pancreatitis via modulation of MAP kinase and MAP kinase phosphatase signaling Am J Physiol Gastrointest Liver Physiol 2008 295 5 G886 94 10.1152/ajpgi.00053.2008 18755806

[B26] Kumar S Novel homologues of CSBP/p38 MAP kinase: activation, substrate specificity and sensitivity to inhibition by pyridinyl imidazoles Biochem Biophys Res Commun 1997 235 3 533 8 10.1006/bbrc.1997.6849 9207191

[B27] Arayatrakoollikit U Pavasant P Yongchaitrakul T Thrombin induces osteoprotegerin synthesis via phosphatidylinositol 3'-kinase/mammalian target of rapamycin pathway in human periodontal ligament cells J Periodontal Res 2008 43 5 537 43 10.1111/j.1600-0765.2007.01071.x 18565131

[B28] Minhajuddin M Protein kinase C-delta and phosphatidylinositol 3-kinase/Akt activate mammalian target of rapamycin to modulate NF-kappaB activation and intercellular adhesion molecule-1 (ICAM-1) expression in endothelial cells J Biol Chem 2009 284 7 4052 61 10.1074/jbc.M805032200 19074768 PMC2640976

[B29] Rahman A Galpha(q) and Gbetagamma regulate PAR-1 signaling of thrombin-induced NF-kappaB activation and ICAM-1 transcription in endothelial cells Circ Res 2002 91 5 398 405 10.1161/01.RES.0000033520.95242.A2 12215488

[B30] Guha M Mackman N The phosphatidylinositol 3-kinase-Akt pathway limits lipopolysaccharide activation of signaling pathways and expression of inflammatory mediators in human monocytic cells J Biol Chem 2002 277 35 32124 32 10.1074/jbc.M203298200 12052830

[B31] Fukao T PI3K-mediated negative feedback regulation of IL-12 production in DCs Nat Immunol 2002 3 9 875 81 10.1038/ni825 12154357

[B32] Yu Y TLR5-mediated phosphoinositide 3-kinase activation negatively regulates flagellin-induced proinflammatory gene expression J Immunol 2006 176 10 6194 201 16670329 10.4049/jimmunol.176.10.6194

[B33] Blum S An inhibitory role of the phosphatidylinositol 3-kinase-signaling pathway in vascular endothelial growth factor-induced tissue factor expression J Biol Chem 2001 276 36 33428 34 10.1074/jbc.M105474200 11445586

[B34] Marra F Involvement of phosphatidylinositol 3-kinase in the activation of extracellular signal-regulated kinase by PDGF in hepatic stellate cells FEBS Lett 1995 376 3 141 5 10.1016/0014-5793(95)01261-0 7498528

